# Lynx web services for annotations and systems analysis of multi-gene disorders

**DOI:** 10.1093/nar/gku517

**Published:** 2014-06-19

**Authors:** Dinanath Sulakhe, Andrew Taylor, Sandhya Balasubramanian, Bo Feng, Bingqing Xie, Daniela Börnigen, Utpal J. Dave, Ian T. Foster, T. Conrad Gilliam, Natalia Maltsev

**Affiliations:** 1 Computation Institute, University of Chicago/Argonne National Laboratory, Chicago, IL 60637, USA; 2 Department of Human Genetics, University of Chicago, Chicago, IL 60637, USA; 3 Department of Computer Science, Illinois Institute of Technology, Chicago, IL 60616, USA; 4 Toyota Technological Institute at Chicago, Chicago, IL 60637, USA

## Abstract

Lynx is a web-based integrated systems biology platform that supports annotation and analysis of experimental data and generation of weighted hypotheses on molecular mechanisms contributing to human phenotypes and disorders of interest. Lynx has integrated multiple classes of biomedical data (genomic, proteomic, pathways, phenotypic, toxicogenomic, contextual and others) from various public databases as well as manually curated data from our group and collaborators (LynxKB). Lynx provides tools for gene list enrichment analysis using multiple functional annotations and network-based gene prioritization. Lynx provides access to the integrated database and the analytical tools via REST based Web Services (http://lynx.ci.uchicago.edu/webservices.html). This comprises data retrieval services for specific functional annotations, services to search across the complete LynxKB (powered by Lucene), and services to access the analytical tools built within the Lynx platform.

## INTRODUCTION

Gaining a greater understanding of molecular mechanisms underlying common multi-gene disorders (e.g. autism, schizophrenia, diabetes) is a major challenge in biomedical research (1). Construction of predictive models of such mechanisms critically depends on the availability of high-throughput genomic data and efficient algorithmic approaches for mining this data with clinical observations and prior knowledge about genotype–phenotype relationships. The unprecedented increase in the production of biological data has led to a number of valuable biological databases. While these databases are very useful in the analysis of data from high-throughput genome-wide associations, expression profiling or next-generation sequencing, accessing these distributed databases can be challenging. Oftentimes, there are a number of databases representing the same classes of information in different non-standard formats with different identifiers and poor cross-references connecting them. The size and frequent changes add to the challenges in using these disparate databases for large high-throughput studies.

To address these challenges, we have developed an integrated Lynx platform that consists of a knowledgebase (LynxKB (2)) which periodically collects various classes of biological data in a structured relational database, analytical tools for the analysis of multi-gene lists and a Lynx web services interface which allows users to query and search LynxKB to retrieve annotations and access the analytical tools. LynxKB integrates many classes of information, including genomic, proteomic, pathways-related, disease-specific, phenotypic, variations, text mining, pharmacogenomics and more from over 35 different public databases (2). It also contains manually curated data collections, including weighted collections of candidate genes extracted from Developmental Brain Disorders Database (DBDB) (3) and LisDB (https://lisdb.ci.uchicago.edu). Currently, Lynx supports only human data (taxonomy: 9606), while mouse and rat related databases are to be integrated in the near future.

Lynx web services are implemented using REST (Representational State Transfer) architecture to provide a simple interface with multiple request types supported (HTTP GET and POST). The results of these RESTful services can be requested in XML and JSON formats for easy consumption.

## ARCHITECTURE, DESIGN AND IMPLEMENTATION

LynxKB is integrated and stored in a normalized relational database using MySQL. In order to connect the information between different sources, appropriate cross-reference data is also integrated. LynxKB currently has a data volume of more than 800GB stored in the database. In order to provide a comprehensive search capability, we use Apache Lucene (http://lucene.apache.org), whereby appropriate indexes of the data are created for Lucene. An advanced search web service is implemented on top of this Lucene framework.

Lynx is implemented using Service-Oriented Architecture concepts, such that all applications within the Lynx framework are built using web services interfaces. Lynx web services are implemented using the Jersey framework (https://jersey.java.net) (JAX-RS Reference Implementation) (4) and Spring framework (5) to provide RESTful web services. The domain specific datatypes and return types for the web services are modeled and represented as XML schemas (XSDs) using JAXB (6) and are automatically translated into domain specific Java objects that are instantiated with data from the MySQL database. Thus, all the domain specific data types used to hold the queried data and return types for web services are defined as XML schemas. As such, the first step in creating any new web service in Lynx starts with defining all of the data types necessary for that service in a XSD. JAXB also ‘marshals’ the java objects back into XML or JSON formats as the web service return types. The XML schemas for return data types help users to implement appropriate client scripts, as they know what data structure to expect from the Lynx web services.

## WEB SERVICES

Lynx provides a large collection of intuitive RESTful web services to get annotations or to perform analyses for a single gene or a list of genes. These web services can be classified into three broad categories: (i) data retrieval services, (ii) search services and (iii) analytical services. All of these web-services in Lynx are genes-centric, such that users can create a list of genes based on certain criteria using the search services, or retrieve annotations and perform analysis on a list of genes. All of these web services can be accessed via HTTP GET or POST based requests and the results can be requested in XML or JSON format.

### Data retrieval services

The Lynx data services allow users to retrieve various classes of annotations (genomic, proteomic, pathways, diseases, phenotypic, toxicogenomic, contextual, interactions, etc.) for a list of genes using Entrez gene IDs or gene symbols. Table 1 below shows a mapping between the specific RESTful resource and sources of the data.

**Table 1.** Lynx data retrieval Web Services

REST resource	Data sources
Geneinfo	NCBI (7), Ensembl (8), UniGene (7), TRANSFAC (9), RefSeq (10)
Pathways	KEGG (11), Reactome (12), NCI (13), BioCarta, Pathway Commons (14)
Diseases	OMIM, AutDB (15), Schizophrenia Gene Resource (SZGR) (16), Diseases (University of Copenhagen), Cancer gene index, DBDB
Interactions	NCBI (7), MINT (17), KEGG , Reactome (12), NCI (13), BioCarta, GeneWays (18)
Tissues	NCBI UniGene (7)
Symptoms	Human Phenotype Ontology (19)

**Figure 1.**
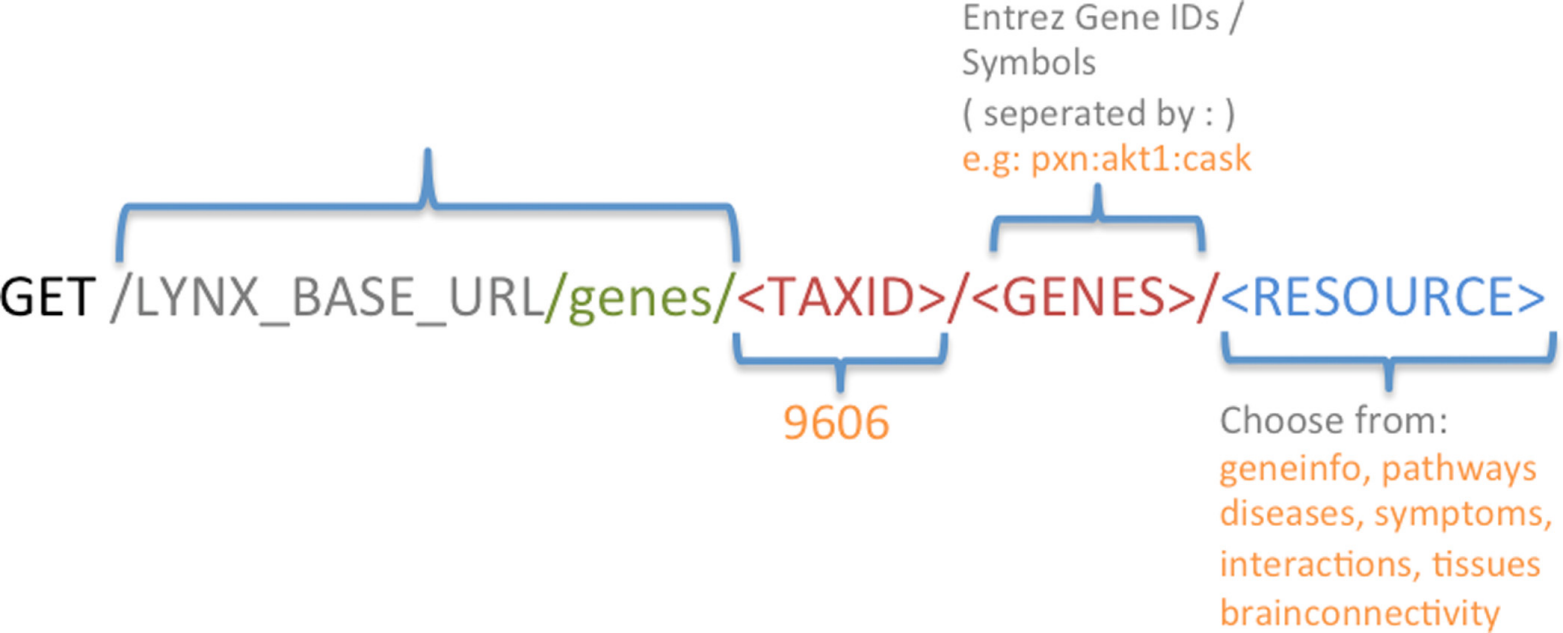
URL Structure to retrieve annotations for a list of genes using HTTP GET.

Figure 1 shows the structure of a URL used for a HTTP GET-based data retrieval web service. Lynx currently supports retrieval of seven features for a given set of genes. For example, the URL: http://lynx.ci.uchicago.edu/gediresources/resources/genes/9606/pxn:akt1:cask/pathways retrieves pathway information from various pathway databases for the three genes (PXN, AKT1, CASK). The web services page on the Lynx website provides some example URLs for retrieving the data. By default, these resources provide the data in XML format. All of these data retrieval web services can be accessed using a POST request as well. For example, the following curl command is a HTTP POST request, equivalent of the HTTP GET request above and returns the pathways in JSON format when run from a Linux terminal or a CURL client program: 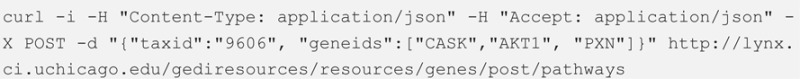


### Search services

Additionally, Lynx provides a robust genes centric search engine. Users can search against any information about genes, pathways, tissues, diseases and symptoms. The queries can be for a specific functional term or general keyword and can request a fuzzy or strict search. Lynx search service also supports Boolean search operators (AND, OR and NOT) allowing multiple search criteria with refined results. The results returned back are genes centric, thus, allowing users to generate gene lists for a given search criterion and use them with other Lynx analytical services as seed genes or test genes. The results also contain detailed annotations for those genes, including relevant pathways, tissues, diseases and symptoms. Currently, Lynx supports keyword-based searches for direct associations of genes with certain features such as diseases, pathways, tissues and symptoms. We plan to implement the searches against ontology-based hierarchies in the future releases.

The following CURL based POST request is an example of a search to get a list of genes that are associated with autism and seizures: 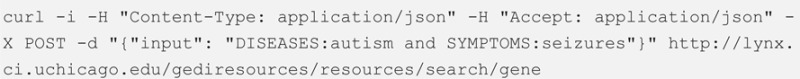


More detailed examples of GET and POST based search services are provided on the Lynx's web services web page.

### Analytical services

Gene lists coming out of high-throughput genomic data analyses (e.g. Next Generation Sequence (NGS) data analysis, gene expression results, Copy-Number Variation (CNV) analysis, expert ranked candidate genes) require subsequent downstream analysis to gain a better understanding of the underlying disease or condition of interest. Here, Lynx's statistical enrichment analysis (20) helps identify the functional categories (e.g. Gene Ontology (GO) terms, diseases, tissues, phenotype, pathways, transcription factor binding sites (21) and enhancers (22)) that are over-represented in the submitted gene set. This enrichment analysis can be performed against all of the human genes or against a specific context (e.g. against genes expressed in a particular tissue or on a particular developmental stage). Lynx's REST based web service can be used to programmatically perform the enrichment analysis by providing the training gene set and the test gene set, selecting the training parameters, *P*-value cutoffs and correction (Bonferroni or False Discovery Rate (FDR)). The example HTTP POST requests shown at http://lynx.ci.uchicago.edu/webservices.html#enrich-genes provide more details on how to access this analytical tool via the REST web service interface. Lynx also provides a tool for network-based gene prioritization for the prediction of high-confidence candidate genes from a large set of genes or even from the entire genome for a disease or phenotype of interest. It is based on PINTA (23,24) and provides five different network propagation algorithms (heat kernel diffusion (25), Page Rank with Priors (26), HITS with prior (27), simple random walk K-step markov (27)) while using STRING version 9.0 (28) as the underlying protein interaction network. Detailed examples on how to use this tool via the REST web service interface are provided at http://lynx.ci.uchicago.edu/webservices.html#nw-prioritization.

## DISCUSSION

In Lynx, we have implemented a large integrated biomedical knowledgebase (LynxKB) and a collection of RESTful web services to access this information in a structured format. In studies involving multi-gene disorders or downstream analysis of next-generation sequence (NGS) data that results in large lists of genes, it tends to be extremely difficult for researchers to accumulate all of the annotation information from multiple sources of databases. Lynx's RESTful web services present a useful one-stop service not only for annotations but also for analysis of unknown lists of genes.

Lynx web services can be used in multiple different scenarios by individual researchers or developers building large systems. For example, individual researchers can use CURL on a command line or write a simple Perl script and collect all of the annotations for their genes of interest. The Lynx web application available at http://lynx.ci.uchicago.edu is a perfect example of a large application that is entirely built on top of Lynx's RESTful services. Similarly, developers can consume these web services within a Next-Generation Sequencing analysis platform such as Galaxy [] by writing tools to annotate the Variant Call Format (VCF) files using Lynx data retrieval services; also, they can analyze the genes in these VCF files using Lynx analytical services to find over-represented pathways, diseases, phenotype and other functional categories.

There are other systems such as David that provide similar web services for annotations and enrichment analysis. In comparison, Lynx's REST based web services provide a comprehensive Lucene based Search service that allows developers to fetch any information at a very granular level. Lynx also allows performing Network based gene prioritization (using five different algorithms) that is a unique capability. Lynx's RESTful architecture makes it easy and flexible in writing client applications in any language of interest and also access the web services from a command-line (using CURL) or in a browser (using GET resources).

In the near future, we will be adding more systems biology related functionalities with emphasis on network analysis and addition of contextual data (e.g. expression data) through out the system. We will also provide ontological support for the user queries. We will include, besides the data retrieval and analysis using already integrated ontologies (e.g. GO) additional ontologies, such as anatomical and developmental ontologies (e.g. developed by Allen Brain Atlas (29), Bgee (30)) as well as additional phenotype and disease-related ontologies and controlled dictionaries (e.g. disease ontology (31), behavioral ontology (https://code.google.com/p/behavior-ontology/)).

We provide detailed documentation with examples (GET and POST request examples) of all of our web services as well as information on any new updates at http://lynx.ci.uchicago.edu/webservices.html. We can be reached via the email address listed on the Lynx website for any specific web service related or general Lynx based support.
